# The association of DNA Repair with breast cancer risk in women. A comparative observational study

**DOI:** 10.1186/1471-2407-12-490

**Published:** 2012-10-22

**Authors:** Jaime Matta, Miguel Echenique, Esperanza Negron, Luisa Morales, Wanda Vargas, Felipe Sánchez Gaetan, Eduardo Ramírez Lizardi, Aníbal Torres, Jose Ortiz Rosado, Guillermo Bolaños, Juan González Cruz, Joaquín Laboy, Ricardo Barnes, Santos Santiago Medina, Ángel Romero, Rosendo Martinez, Julie Dutil, Erick Suarez, Carolina Alvarez-Garriga, Manuel Bayona

**Affiliations:** 1 Department of Pharmacology, Physiology, and Toxicology, Department of Surgery, Ponce School of Medicine and Health Sciences, Ponce, Puerto Rico, 00732; 2 Auxilio Mutuo Hospital, San Juan, Puerto Rico; 3 Department of Surgery and Damas Hospital, Ponce School of Medicine and Health Sciences, Ponce, Puerto Rico; 4 Private Practice, Ponce, Puerto Rico; 5 Department of Surgery, Ponce School of Medicine and Health Sciences, Ponce, Puerto Rico; 6 Department of Surgery and Private Practice, Ponce School of Medicine and Health Sciences, Ponce, Puerto Rico; 7 Department of Surgery and San Lucas Hospital, Ponce School of Medicine and Health Sciences, Ponce, Puerto Rico; 8 Outpatient Clinics, Ponce School of Medicine and Health Sciences, Ponce, Puerto Rico; 9 Dr. Pila Hospital, Ponce, Puerto Rico; 10 Department of Biochemistry, Ponce School of Medicine and Health Sciences, Ponce, Puerto Rico; 11 School of Public Health, University of Puerto Rico, Medical Sciences Campus, San Juan, Puerto Rico; 12 Public Health Program, Ponce School of Medicine and Health Sciences, Ponce, Puerto Rico; 13 Current address: Division of Epidemiology/OSB/CDRH, Food and Drug Administration, Silver Spring, Maryland, USA

**Keywords:** Breast cancer, DNA repair capacity, Association, Risk, Biomarker

## Abstract

**Background:**

Previous studies have found a link between a low DNA repair capacity (DRC) level and increased cancer risk. Our aim was to assess the statistical association of DRC level and breast cancer (BC) using a case–control epidemiological study in a Hispanic community.

**Methods:**

We conducted a comparative observational study to assess the validity of DRC in detecting BC in 824 women throughout Puerto Rico. Over a 6-year period, we compared 285 women newly diagnosed with BC to 539 without BC. DRC levels were measured in lymphocytes by means of a host-cell reactivation assay. We assessed the sensitivity, specificity, and association using the receiver operating characteristic curve analysis. Multiple logistic regression-adjusted odds ratios were estimated with 95% confidence level to measure the strength of the association of DRC and BC after adjusting for all confounders simultaneously.

**Results:**

Compared to women without cancer, women with BC showed an average decrease of 60% in their DRC levels (*p* < 0.001). Validity of the association of DRC as a measure of BC risk showed a sensitivity of 83.2% and specificity of 77.6% (*p* < 0.0001).

**Conclusions:**

Our results support the usefulness of DRC level as a measure of BC risk. Additional studies in other populations are needed to further verify its usefulness.

## Background

Breast cancer (BC) is the most common cancer worldwide affecting women, accounting for 20% of all malignancies in females [1]. In 2010, an estimated 207,090 U.S. women were diagnosed with BC [2]. In Puerto Rico, BC accounted for 30% of all cancers in 2009. This percentage represented 1,776 new patients, which was the highest incidence per organ (Cancer Registry of Puerto Rico). Despite the declining trend in mortality, BC is currently the second leading cause of cancer death in women living in the U.S. and the first cause of cancer mortality in Puerto Rican women [3]. Even with the many advances made in diagnostic procedures and treatments for BC, early-detection methods are needed.

Because age is a principal risk factor for cancer, studies on aging have provided additional understanding of DNA repair processes [4]. However, aging is not the only cause of genomic instability that can lead to cancer. Individuals vary in their inherent sensitivities to mutagens and carcinogens due to differences in their DNA repair capacity (DRC) levels [5]. Several studies have shown that lower DNA repair capacity correlates with higher cancer risk [6-18]. Epidemiological studies using functional repair assays in lymphocytes have also demonstrated that DRC varies greatly among individuals, and that a low DRC is a significant risk factor for the development of several types of cancer [9,12,14,17,19-21].

With respect to DNA repair in cases of BC, the Nucleotide Excision Repair (NER) pathway is receiving increasing attention [22]. Numerous studies using lymphocytes have demonstrated an association between BC incidence and NER deficiency [11,23-28]. Collectively, they suggest that NER deficiency may contribute to the etiology of sporadic and familial BCs. If we can learn more about that association and develop a readily available clinical method for detecting DRC, then we can identify, treat, and arrest BCs earlier.

The aim of our study was to to assess the statistical association of DRC level and breast cancer (BC) using a case–control epidemiological study in a Hispanic community. In contrast to previously published studies, including our (Ramos *et al.* 2004), we used a large sample size (824 participants) for increased statistical power. This clinic-based, observational study [29] compared recently diagnosed, treatment-naïve, histopathologically confirmed BC patients to women without BC.

## Methods

### Epidemiologic design

Over a 6-year period, we recruited 824 women of Hispanic origin, age 21 or older: 285 women newly diagnosed with BC and 539 without BC. Calculations of sample size done initially revealed that a sample size of 824 participants (312 women with BC, 515 women without BC) would allow us to have a statistically significant odds ratio as low as 1.7 when the percent exposed to a low DRC among controls is 15% or higher (e.g., 15% controls are 21 to 30 years of age) with 5% significant level and 80% of statistical power.

The population represents a genetically diverse population that is an admixture of European, African, and Amerindian ethnic groups (per studies from 106 ancestry markers—Dr. Julie Dutil, unpublished observations). Women who were obtaining mammograms and other routine gynecological and primary care/screening services at the same medical offices where patients with BC were being treated were recruited consecutively at the following locations: Ponce School of Medicine and Health Sciences Outpatient Clinic, Auxilio Mutuo Hospital (San Juan), Damas Hospital (Ponce), and St. Luke’s Hospital (Ponce), as well as Yauco and other selected collaborating cities throughout Puerto Rico, representing 65 (83%) of the 78 municipalities (counties) on the island. Because Puerto Rico offers universal health insurance coverage, any healthy women who might develop BC would be treated in the same facilities where BC patients were recruited. This selection procedure minimized selection bias due that could have otherwise been a factor (site, screening/treatment modalities) if healthy women were recruited from the general population by other means (e.g., through random-digit dialing, as noted by Rothman *et al.*[30]).

This study was approved by the IRB of the Ponce School of Medicine and Health Sciences (Ponce, Puerto Rico) and participating hospitals. Informed consent was obtained from all participants before interviewing them, drawing blood samples, collecting tumor material, and obtaining pathology reports.

The two main inclusion criteria for selecting women without BC were 1) a normal breast examination done by a primary care physician and 2) a normal mammogram, both within the last six months. These criteria reduced the likelihood of the existence of undiagnosed BC in this cohort.

We studied only recently diagnosed, treatment-naïve BC patients with primary tumors. Exclusion criteria included those with metastatic BC, secondary BC, breast metastases from another type of cancer, or any acquired or genetic immunodeficiency. Because blood transfusions, chemotherapy, and radiotherapy can significantly affect DRC [31-33], patients who had received any of those treatments in the past 5 years were also excluded from the study.

Pathology reports from all patients were obtained to confirm the 1) diagnosis, 2) tumor grade, 3) tumor size, 4) presence or absence of axillary lymph node metastasis, and 5) other clinically relevant information. An epidemiological questionnaire soliciting information and variables related to BC risk was provided to each participant.

### Blood collection and isolation of lymphocytes from women

Approximately 30 mL of peripheral blood was obtained from each participant and stored in heparinized tubes. The lymphocytes were then isolated by the Ficoll gradient technique and suspended in 2 mL of freezing media containing 10% dimethyl sulfoxide, 40% RPMI 1640 medium, 50% fetal bovine serum, and 1% antibiotic/antimycotic. Aliquots were stored in a −80°C freezer for 1–3 weeks. The lymphocytes were later thawed in batches of 5–7 samples for the host-cell reactivation (HCR) assays (details follow). Collection periods were approximately the same for patients and women without BC because recruitment was conducted concurrently.

### Plasmid preparation for host cell reactivation (HCR) assay for measuring DNA repair capacity

The late Dr. Lawrence Grossman (Johns Hopkins School of Public Health, Baltimore, MD) provided the luciferase plasmid for the HCR assay and the protocol for its use. A non-replicating plasmid expression vector (pCMVl*uc*) of 4,863 base pairs was genetically engineered to contain a bacterial luciferase reporter gene that is not present in a mammalian cell. The gene was damaged by ultraviolet C radiation (254 nm) exposure in a controlled, quantitative manner (dose–response curve) so that the level of its expression was a direct measure of the repair capacity of the host mammalian cell. The plasmid construct containing the luciferase gene (LUC) was irradiated at 0, 350, and 700 J/m2 using a 254-nm UVC lamp [11]. This plasmid construct and its validation have been described previously [34]. The controlled, quantitative UV exposure produced a dose–response curve so that the level of its expression was a direct measure of the repair capacity of the host mammalian cell.

### HCR assay to measure DRC

The HCR assay that we utilized to measure DRC levels in lymphocytes has been described in previously published molecular epidemiological studies of cancer [9,11,12,34-37]. This assay measures the total DRC of transfected lymphocytes. Results reflect the host cells’ overall repair capacity, although HCR primarily detects activity of the nucleotide excision repair (NER) pathway [38].

To assess this assay’s precision, we used a data subset that involved duplicates of 50 women with BC, 50 women without BC, and 90 samples of three commercial cell lines. A correlation of 0.97 (95% overall confidence interval: 0.95–1.00) was found (*P* < 0.001; data not shown). In addition, we repeated the assay if we found any inconsistencies between duplicates.

The measurement of DRC has a coefficient of variation of 23% in the data presented in this study. A batch effect associated with inter-technician variability in performance of the DRC assay was found; this was corrected by excluding 36 samples (35 BC cases, 1 control). Grossman and Wei [5] demonstrated that, at this precision, our assay can distinguish both intra- and inter-assay variation by being able to maintain the ranks of DRC values in samples measured in triplicate from multiple patients.

### Validation of stable transfection

To confirm achievement of stable transfection, we utilized the Dual-GloR® Luciferase Assay System (Promega; Madison, WI), which is based on the combined use of the Firefly and *Renilla* luciferases proteins as co-reporters. The assay allows for analysis of mammalian cells (e.g., lymphocytes) containing genes for Firefly and *Renilla* luciferases grown as positive controls. To determine whether our results would vary significantly between cryopreserved versus fresh blood samples, we took dual samples from 5 patients (total of 10 blood samples) and assayed 5 immediately after phlebotomy (fresh), then cryopreserved the other 5 samples at −80°C and analyzed those several weeks later.

### Preparation of samples and controls

Previously frozen peripheral blood lymphocytes from patients and women without BC were assayed in batches, as described by Ramos *et al.*[11]. Peripheral blood lymphocytes with >95% viability were incubated for 72 h with phytohemagglutinin and then were transfected with undamaged or damaged plasmid DNA. Cells isolated from xeroderma pigmentosum patients corresponding to complementation groups C and D (XPC, XPD) were used as positive controls (cell lines GM 02246D and GM 02253F, respectively; Coriel Institute Medical Research; Camden, NJ).

### Calculation of DRC

The assay for the gene expression of luciferase activity was measured using a luminometer (Turner Designs, model TD-20/20, Sunnyvale, CA). The percentage of DRC was calculated as the percentage of luciferase activity present after the repair of damaged plasmid DNA, compared to the DRC of undamaged plasmid DNA (100%). This method produced a range of DRC values from nearly 0 to 19% DRC.

### Statistical analyses

The association of DRC levels was based on the accuracy detecting BC when a woman has a high versus low DRC level. Data analyses were conducted by using the IBM SPSS statistical package version 17.0 (IBM; Armonk, N.Y.). The Wilcoxon or Mann Whitney *U*-test for independent samples was used to assess the statistical significance of the mean difference to account for non-normally distributed variables such as in DRC [39].

During crude analysis, variables such as DRC, age, weight, and body mass index, were first analyzed as continuous variables. The mean difference was used to assess DRC and anthropometric difference between patients with BC and women without BC using the logistic regression model [39]. After this step, for stratified analysis, continuous variables were categorized by using percentiles as cutoff points [29]. DRC was divided in low and high DRC using the median from the whole sample, also to divide on low, medium and high DRC, terciles were used from the whole data. Stratified analyses were conducted to identify and assess potential confounders or interaction effects of the association between BC and DRC [29,30]. After stratified analyses, a multivariable logistic regression model was used, odds ratio was estimated with 95% confidence level to assess the strength of the association between DRC level and BC adjusting for potential confounders [30].

## Results

### Association between DNA Repair capacity levels and population variables in women with and without breast cancer

**Figure 1:**
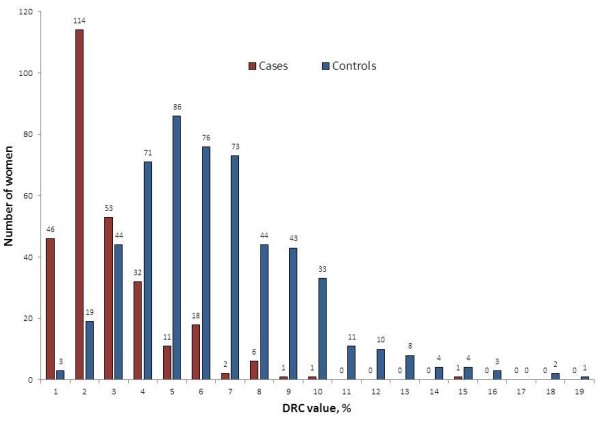
**% DRC of female cohorts in Puerto Rico with breast cancer (n = 385) and without breast cancer (n = 539).** DRC was measured in lymphocytes by a host reactivation assay with a luciferase reporter gene. Women with breast cancer have a DRC that, on average, is 60% lower than women without breast cancer (*P* < 0.001, Mann Whitney *U*-test).

**Figure 2:**
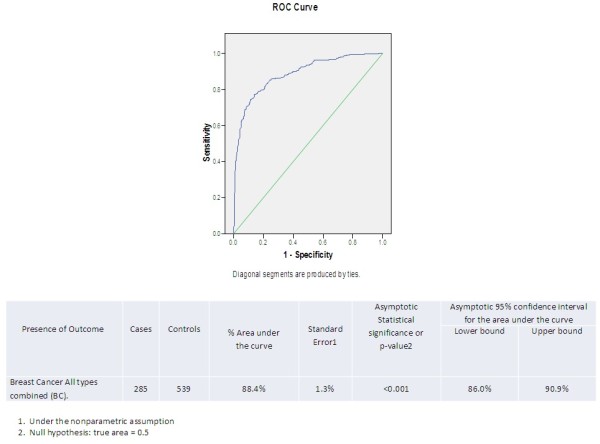
DRC detecting the presence of breast cancer ROC Curve (285 cases and 539 Controls).

Women with BC (n = 285) had a mean DRC of 2.40%; this was 60% lower than the average DRC level (*P* < 0.001, Mann Whitney *U*-test) than in the 539 women without BC, they had a mean DRC level of 6.13% (Figure 1). For every 1% decrease in DRC, there was 64% greater likelihood of having BC. These findings were statistically significant (p<0.05). ROC curve analysis [40,41] was used to assess the accuracy of detecting BC with DRC [42]. The area under the ROC curve was found to be 88.4% (95% CI: 86%, 90.9%), and the 3.7% DRC cutoff point reached 83.2% sensitivity and 77.6% specificity (Figure 2).

Table 1 presents the crude and multivariate adjusted odds ratios to determine the strength of the associations between BC and other selected variables. Different blocks of variables were tested to find the best model for adjusting for potential confounding. The model that was finally used included, age, body-mass index, marital status, number of children, family history of BC, and smoking. The odds of having BC increased 2% with every year of age (95% CI: 1.01, 1.05). A family history of BC increased the odds of having BC by 1.7 times (95%CI: 1.0, 2.8). Interesting but statistically not significant findings included the following: the odds of having BC increased 10% with the birth of each child, 3% by each percent increase in BMI, 40% by history of pregnancy and 10% by smoking, also, for breastfeeding or alcohol intake; those decreased the odds for BC by 20%, respectively. Compared to married women, widows had 4.6 times higher odds of having BC (95% CI:1.6, 13.7); single women had 5.9 times higher odds of having BC (95% CI: 2.3, 15.6), and divorced had 3.6 times more likelihood of having BC (95% CI: 1.2, 10.5).

**Table 1:** Odds ratio estimation to assess the association between breast cancer (BC) and DNA repair capacity (DRC) and other selected variables in women with BC (n=285) and women without BC (n=539)

**Variable**	**Women with BC n (mean)**	**Women without BC n (mean)**	**Crude OR**^**1**^	**Adjusted OR**^**2**^	***P*value**
			**(95% CI)**	**(95% CI)**	
Age (continuous)*	285 (56.7)	539 (52.4)	1.02 (1.02,1.04)	1.02 (1.01, 1.05)	0.001
Number of children	273(2.4)	517 (2.1)	1.2 (1.1, 1.3)	1.1 (1.0, 1.3)	0.055
# Missing (34)					
Increasing BMI (continuous)*	284 (27.8)	534 (27.2)	1.02 (0.9, 1.1)	1.03 (1.0, 1.1)	0.118
# Missing (6)					
Family history of BC					
Yes	64	78	1.7 (1.2, 2.5)	1.7 (1.0, 2.8)	0.054
No	221	461			
Marital status					
Married	154	362	Referent	Referent	
Single	61	92	4.2 (2.2, 7.8)	5.9 (2.3, 15.6)	<0.001
Divorced	40	66	2.7 (1.4, 5.3)	3.6 (1.2, 10.5)	0.025
Widow	30	17	2.9 (1.4, 5.9)	4.6 (1.6, 13.7)	0.006
# Missing (2)					
Irregular menstrual cycles					
Yes	183	320	1.2 (0.9,1.6)	1.4 (0.8, 2.2)	0.207
No	102	213			
# Missing (6)					
History of pregnancy					
Yes	220	401	1.2 (0.8,1.6)	1.4 (0.8,2.2)	0.382
No	65	138			
History of breastfeeding					
Yes	122	246	0.8 (0.6, 1.1)	0.8 (0.5, 1.2)	0.256
No	118	194			
# Missing (144)					
Alcohol intake					
Yes	42	98	0.8 (0.6, 1.2)	0.8 (0.5, 1.7)	0.526
No	243	441			
Smoking					
Yes	37	48	1.6 (0.9, 2.4)	1.1 (0.6, 2.1)	0.707
No	248	491			

*Mean values presented in parenthesis.

Comparisons were measured with the crude^1^ and the adjusted^2^ multiple logistic regression odds ratio (OR)^3^ in the total sample divided by patients and women without breast cancer.

^1^Crude or unadjusted analysis: The analysis is carried out without taking into consideration potential confounders.

^2^Adjusted analysis: The analysis is carried out adjusting for the potential effect of all confounding variables simultaneously by using logistic regression model. The associations were adjusted by DRC, age, body mass index, family history of breast cancer, number of children, marital status, and/or smoking.

Table 2 presents the crude and multivariate adjusted associations between BC and DRC. When DRC was analyzed as a continuous variable the adjusted odds ratio was 2.3 (95% CI: 2.0, 2.6) (*P* < 0.001). The associations between BC and DRC, both as a continuous and as a categorical variable, became stronger after multivariate adjustment (*P* < 0.001). The DRC in three categories levels showed a strong association with BC and a statistically significant linear trend (*P* < 0.001). Women with low DRC levels (≤ 3.0%) had 60.6 (95% CI: 32.0, 114.6) times the odds of having BC as women with high DRC levels (≥ 5.81%) when adjusting for potential confounders.

**Table 2:** DNA Repair Capacity (DRC) of women with breast cancer (BC) and women without BC by age group

**Variable**	**Women with BC n (mean DRC)**	**Women without BC n (mean DRC)**	**Crude OR**^**1**^	**Adjusted OR**^**2**^	***P*value**
			**(95% CI)**	**(95% CI)**	
Decreasing DRC (continuous)*	285 (2.41)	539 (6.13)	2.2 (1.9, 2.4)	2.3 (2.0, 2.6)	< 0.001
DRC					
Low (< 4.3%)	246	168	13.9 (9.4, 20.4)	15.1 (10.0, 22.9)	< 0.001
High (≥ 4.3%)	39	371			
DRC levels^3^					
Low (0.0-3.0%)	213	66	54.6 (30.3, 98.5)	60.6 (32.1, 114.6)	< 0.001
Medium (3.01-5.8%)	57	219	4.4 (2.4, 8.0)	13.0 (8.5, 20.0)	< 0.001
High (≥5.81%)	15	254	Referent	Referent	

*Mean values presented in parenthesis.

Comparisons were measured with the crude^1^ and the adjusted^2^ multiple logistic regression odds ratio (OR) in the total sample divided by patients and women without breast cancer.

^1^Crude or unadjusted analysis: The analysis is carried out without taking into consideration potential confounders.

^2^Adjusted analysis: The analysis is carried out adjusting for the potential effect of all confounding variables simultaneously by using logistic regression model.

^3^Chi-Square for linear trend = 233.6, *p*< 0.001.

## Discussion

While the HCR assay as described in this study assesses the overall DNA repair capacity, it preferentially measures repairs made by the nucleotide excision repair (NER) pathway. NER repairs bulky adducts caused by UV irradiation [38] and many carcinogens such as the ones contributing to carcinogenesis presented by BC patients [5-8]. Low levels of NER repair have been detected in peripheral blood lymphocytes and tumor samples of BC patients [5-8]. In addition, exposure to ionizing radiation is a risk factor for BC [9]. DNA lesions arising from ionizing radiation and many carcinogens causing bulky lesions are repaired by NER and deficiencies in the NER pathway can increase genomic instability.

The HCR assay, developed in the early 1990s, provided a way to obtain a quantitative, phenotypic measurement of DRC in lymphocytes [5]. The subsequent development of a plasmid based on luciferase activity instead of radioactivity resulted in stable transfection efficiencies [38]. Because of the high cost of this assay, its technical complexity, and its labor intensiveness, relatively few laboratories in the world currently utilize it for large-scale molecular epidemiology studies of cancer [9,11,14,15,17,36,37,43].

Studies published to date of HCR assays of lymphocytes have frequently, but not always [34], found a link between a low DRC and increased cancer risk. Our group [11] was the first to report that a low DRC was a risk indicator for BC. That initial DRC study consisted of a small sample size (n = 36 patients) and did not have the benefit of ROC analysis or a well-defined percent DRC level (e.g. low, medium, high) as a BC risk estimator compared to the analyses presented in this study. In the current study, we furthered classified DRC into terciles; low, medium and high levels. This methodology has the potential to serve as a biomarker of risk of BC (United States Patent Trade Office US 8,163,473 B2). Our adjusted OR (2.3) for DRC as a continuous variable is within the range of similar ORs values for published studies for basal cell carcinoma skin cancer (Wei *et al.* 1993), lung cancer (Wei *et al.* 2000), head and neck cancer (Cheng *et al.* 1998), prostate cancer (Hu *et al.* 2004) and BC (Shi *et al.* 2004) [12,26,44,45].

However, when DRC was analyzed either as a dichotomous or as a categorical variable (low, medium, high), the ORs are much higher. Such high ORs are unusual and have been reported only a few studies. For example, Latimer *et al.* (2010) recently estimated that the sensitivity of detecting tumors based on reduced NER levels alone is 95%, the specificity is 74%, and the odds ratio is 53.8 (95% CI, 28.3–102.4). This group demonstrated the critical importance of the NER pathway in BC by measuring the NER capacity in breast tumors (n=19) and normal primary cultures expressed relative to the mean of these normal BC tissues. The mean NER capacity of the tumor samples was significantly lower than that of normal breast tissues, averaging only 44% of normal activity (*P* < 0.001). Some other studies have also reported very high ORs when strong associations are found. This has occurred with the association of cervical cancer with HPV exposure. Some HPV studies have reported very powerful associations. For example, a case control study for HPV by Powell *et al.* (2011) reported ORs as high as 2770; Chen *et al.* (2011), as high as 75; Chuang *et al.* (2012), ORs 18.1 and higher; and Almonte *et al.* (2011), ORs of 16.1 [46-49].

In this study, we observed an unusually strong increment on the OR after adjustment. Therefore, we repeated the analysis entering, first entering DRC alone in the model; then the covariates were entered one by one. In doing that, we found that the adjusted OR increased gradually as we added each covariate to the model.

No evidence of interaction terms were found in the multivariate logistic model (p>0.05), so DRC can be considered as a potential independent risk factor for DRC because it is biologically plausible, shows a strong association, and exhibits a dose–response relationship after adjusting for all confounders simultaneously.

Despite the usefulness of the HCR assay to estimate DRC and detect BC risk, future prospective studies are needed to further validate the use of this approach. Specifically, further studies are needed to ascertain 1) whether women without BC with a low DRC have a higher incidence of BC compared to those who have a high DRC and 2) whether women with BC and a low DRC are at an increased risk of recurrent BC compared to those with high DRC.

In this study, patients were evaluated for DRC after BC was diagnosed; thus, reverse causation could be present if BC lowers DRC, which would explain why we found a low DRC associated with BC. Nevertheless, reverse causation is an unlikely explanation of our findings because experimental evidence demonstrates that a low DRC or impairments in DNA repair pathways and/or genes is a mechanistic component involved in BC carcinogenesis [2,11,28,50,51] or relapse [24] for the transition to hormone independence [52].

Given that newly diagnosed patients are not always truly incident patients, we compared DRC results to tumor size (data not shown) as a surrogate for the length of time period between disease onset and diagnosis to address the potential changes that DRC in lymphocytes might have had after a woman develops BC. With these analyses, we did not find any statistically significant DRC level differences by tumor size that could interfere with our comparisons.

DRC at its cutoff point selected based on its median value (4.3%) was found in this study to have 83.2% sensitivity and 77.6% specificity for detecting BC. ROC analysis showed a high accuracy detecting BC (88.4% of the area under the curve), which was statistically significant. Because any given specific value of DRC level is associated with a quantitative BC risk estimate, DRC has the potential to be a biomarker to rule out BC risk in low-prevalence or low-risk populations or confirm its presence in high-prevalence or high-risk populations. However, a higher specificity and sensitivity would increase the value of DRC as a detector of BC. In addition, these findings need to be validated in other populations and with future prospective studies.

## Conclusions

Our findings confirm the hypothesis that a low DRC is an important risk factor for BC. Thus, measuring DRC could be a useful tool to assess BC risk, especially in women from low-prevalence or low-risk populations, such as the population that we studied. For every percent unit of decrease in DRC, there is 64% more likelihood of having BC. Although our results support the validity of using DRC level to estimate BC risk, further studies (including prospective studies) are needed to verify whether such an appraisal would be a useful addition to existing screening and risk-assessment tests for BC, as other covariates (for example, family history, age at first pregnancy) could modify its predictive value.

## Abbreviations

BC: Breast cancer; DRC: DNA repair capacity; HCR: Host cell reactivation assay; LUC: Luciferase; NER: Nucleotide Excision Repair.

## Competing interest

Two of the authors (JM, MB) were awarded have received a utility patent award (US 8,163,473 B2) from the United States Patent Trade Office for some findings presented in this manuscript.

## Authors' contributions

The experiments were performed by EN, LM. Participant recruitment and administration of epidemiological questionnaire was done by WV. JD explained DRC results to some participants, performed ancestry marker analysis and helped with DRC assay internal validation. Participant clinical samples were provided by ME, FSG, ERL, AT, JOR, GB, JGC, JL, RB, SSM, AR and RM. Study design, supervision and preparation of manuscript was done by JM. Epidemiological and biostatistical analysis was done by MB, ES and CA. Manuscript preparation was done by JM and MB. All authors read and approved the final manuscript.

## Disclaimer

The opinions and assertions presented in this article are the private views of the authors and are not to be construed as conveying either official endorsement or criticism by the US Department of Health and Human Services, the Public Health Service, or the US Food and Drug Administration.

## Pre-publication history

The pre-publication history for this paper can be accessed here:

http://www.biomedcentral.com/1471-2407/12/490/prepub
